# A Systematic Review of Metabolomic Biomarkers for the Intake of Sugar-Sweetened and Low-Calorie Sweetened Beverages

**DOI:** 10.3390/metabo11080546

**Published:** 2021-08-19

**Authors:** Samuel Muli, Jantje Goerdten, Kolade Oluwagbemigun, Anna Floegel, Matthias Schmid, Ute Nöthlings

**Affiliations:** 1 Nutritional Epidemiology, Department of Nutrition and Food Sciences, University of Bonn, Friedrich-Hirzebruch-Allee 7, 53115 Bonn, Germany; koluwagb@uni-bonn.de (K.O.); noethlings@uni-bonn.de (U.N.); 2 Department of Epidemiological Methods and Etiological Research, Leibniz Institute for Prevention Research and Epidemiology—BIPS, Achterstr. 30, 28359 Bremen, Germany; goerdten@leibniz-bips.de (J.G.); floegel@leibniz-bips.de (A.F.); 3 Institute for Medical Biometry, Informatics and Epidemiology (IMBIE), University Hospital Bonn, Venusberg Campus 1, 53127 Bonn, Germany; matthias.schmid@imbie.uni-bonn.de

**Keywords:** sugar-sweetened beverages, low-calorie sweetened beverages, metabolomics, biomarkers

## Abstract

Intake of added sugars (AS) is challenging to assess compared with total dietary sugar because of the lack of reliable assessment methods. The reliance on self-reported dietary data in observational studies is often cited as biased, with evidence of AS intake in relation to health outcomes rated as low to moderate quality. Sugar-sweetened beverages (SSBs) are a major source of AS. A regular and high intake of SSBs is associated with an overall poor diet, weight gain, and cardiometabolic risks. An elevated intake of low-calorie sweetened beverages (LCSBs), often regarded as healthier alternatives to SSBs, is also increasingly associated with increased risk for metabolic dysfunction. In this review, we systematically collate evidence and provide perspectives on the use of metabolomics for the discovery of candidate biomarkers associated with the intake of SSBs and LCSBs. We searched the Medline, Embase, Scopus, and Web of Science databases until the end of December 2020. Seventeen articles fulfilled our inclusion criteria. We evaluated specificity and validity of the identified biomarkers following Guidelines for Biomarker of Food Intake Reviews (BFIRev). We report that the ^13^C:^12^C carbon isotope ratio (δ^13^C), particularly, the δ^13^C of alanine is the most robust, sensitive, and specific biomarker of SSBs intake. Acesulfame-K, saccharin, sucralose, cyclamate, and steviol glucuronide showed moderate validity for predicting the short-term intake of LCSBs. More evidence is required to evaluate the validity of other panels of metabolites associated with the intake of SSBs.

## 1. Introduction

Added sugar (AS) refers to sugars, syrups, or caloric sweeteners added to foods during preparation, processing in the industry, or by consumers at the table [1]. A high intake of AS is a public health concern, because of its associated health risks. The World Health Organization (WHO) recommends less than 10% of the total daily energy intake from free sugars, which includes AS and sugars naturally present in honey, syrups, fruit concentrates, and juices [2]. In reference to a total energy intake of 2000 kcal per day, the WHO recommendation corresponds to 50 g of free sugars [3]. Despite the imprecise definition of AS and free sugars in epidemiologic studies, there is consensus that consumption significantly exceeds WHO recommendations. In a German cohort study, the median intake of AS ranged between 11.6% and 13.3% and free sugars between 15.2% to 17.5% in children and adolescents aged 3 to 18 years [4]. In the USA, a national survey reported a mean adjusted estimate of AS intake in children aged 2–18 years as 14% of their daily energy intake [5]. 

A major source of dietary AS is sugar-sweetened beverages (SSBs). A regular and high intake of SSBs is associated with overall poor diet quality [6], weight gain and progression of obesity [7], increased risk for diabetes [8], cardiovascular diseases [9,10,11], and a low-grade inflammatory state [12,13]. Low-calorie sweetened beverages (LCSBs), which contain non-nutritive sweeteners, are commonly marketed as healthier alternatives to SSBs [14]. However, emerging evidence from observational studies suggests their inverse association with cardiometabolic health [15], including risk for ischemic stroke and all cause dementia [16], impaired insulin sensitivity in healthy individuals [17], and increased risk for cardiovascular diseases [18]. A causal link between SSBs/LCSBs intake and negative health effects is yet to be established. 

As all consumed foods like SSBs/LCSBs are metabolized, their metabolites could be a window to their intake and may also improve our understanding of the causal link with the aforementioned health conditions. This is especially important because varying opinions persist about evidence from self-reported dietary assessment tools, such as dietary food records, 24-h dietary recalls, and food frequency questionnaires (FFQs). These instruments are often cited as having inherent biases of recall and misreporting, which may lead to incorrect estimations of the associations between intake and health outcomes [19]. Evidence from studies suggest that foods considered socially undesirable, such as those high in AS like SSBs, are mostly underreported [20,21]. In part, such challenges have continued to fuel the debate on the validity of the associations between the intake of SSBs/LCSBs and health risks. This potentially undermines public health messages that urge the public to reduce the intake of AS and SSBs. Objective biomarkers for the dietary intake of SSBs/LCSBs could aid in overcoming this longstanding challenge by complementing the existing dietary instruments to strengthen the evidence on connection between intake and health status [22]. 

The discovery and validation of biomarkers of SSB and LCSB intake remains a high-priority research area, with rapidly growing evidence of dietary signatures in blood, urine, fingernails, hair, and other human tissues [23]. Some biomarkers have been proposed through targeted metabolomics methods, and have been validated in small, controlled feeding studies. However, untargeted methods of biosamples following exposure to SSBs and LCSBs have also produced panels of novel metabolites that need further investigation and validation. Therefore, this study collates the latest evidence from studies applying metabolomics methods for the discovery of candidate biomarkers associated with the intake of SSBs and LCSBs. 

## 2. Materials and Methods

### 2.1. Literature Search

To identify the biomarkers of food intake (BFI) for SSBs and LCSBs, an extensive literature search was conducted following the Guidelines for Biomarker of Food Intake Reviews (BFIRev) [24] and the PRISMA statement for systematic reviews [25], whenever meaningful. We registered the review project with the OSF Registries (DOI: 10.17605/OSF.IO/97VFY, https://osf.io/2pvr3/, accessed on 19 August 2021). We comprehensively searched four electronic databases, Medline, Embase (in OVID SP), Scopus, and Web of Science, using the following search terms, adapted appropriately to each database: (sugar*sweet*beverage* OR SSB* OR beverage* OR added sugar* OR caloric*sweet* OR soda* OR diet*beverage* OR soft drink* OR low*calorie*sweet*beverage* OR LCSB* OR artificial*sweet*beverage* OR ASB* OR fruit flavored drink* OR carbonated drink* OR juice*) AND (biomarker* OR marker* OR metabolite* OR metabolom* OR biomonitor* OR biosignature* OR bioavailability) AND (intake OR diet OR dietary pattern* OR dietary habit* OR eating pattern* OR food* OR meal* OR nutrition*assessment OR nutrition* survey*) AND (plasma OR urin* OR serum OR blood OR hair). The search was limited to papers published on human studies and in English, from inception dates until the end of December 2020. Studies on animal models were excluded. We used EndNote (version X9) and Rayyan QCRI programs for reference management and abstract screening, respectively. Two independent reviewers (S.M. and J.G.) screened all the titles and abstracts, and conducted full text reading and subsequent data extraction. 

### 2.2. Evaluation of Specificity of Identified Biomarkers 

We performed an extensive literature search, as recommended in the BFIRev guidelines [24], to evaluate the specificity of the identified candidate biomarkers. In a second search step, we evaluated the specificity of the candidate biomarkers in the Human Metabolome Database (HMDB), the Food Database (FooDB), and the Phenol-Explorer. If the reported compound was identified as a biomarker for non-SSBs and non-LCSBs food items, such a compound would be removed from further selection. Next, to confirm if the identified potential biomarkers were detected in other foods, further literature search was performed: (“name and synonyms of biomarker candidate”) AND (biomarker* OR marker* OR metabolite* OR metabolom* OR biomonitor* OR biosignature*), which was executed on the Google Scholar search engine. Compounds present in other foods were determined as lacking specificity for SSBs or LCSBs. 

### 2.3. Evaluating of Validity of Biomarkers

We adopted the framework proposed by Dragsted et al. [26] to assess the validity of the identified biomarkers of SSBs and LCSBs. This framework provides eight groups of validity criteria for assessing the validation and application of BFIs, namely, plausibility, dose−response relationship, time−response (single-meal time response and repeated intakes), robustness, reliability, stability, analytical performance, and reproducibility. In total, the validity of the candidate biomarkers was assessed by answering nine questions, with either a yes, no, or uncertain/unknown. Selected biomarkers were then graded, with the scores reflecting the current validity rating of the biomarker as informed by available evidence. 

### 2.4. Evaluating Quality of Evidence

Because of the lack of standard validated tools for evaluating the quality of evidence of the metabolomics studies, we applied two assessment tools to assess the risk of bias and biomarker measurement characteristics. For quality assessment of the evidence (i.e., risk of bias and study quality), we applied the NutriGrade scoring system, which uses the Grading of Recommendations, Assessment, Development, and Evaluations (GRADE) approach [27]. To evaluate the quality of the biomarker measurement, we applied the Biomarker-based Cross-sectional studies (BIOCROSS) evaluation tool, which is especially adapted for biomarker measurement, representing biosample and assay methods, laboratory measurement, and biomarker data models [28]. 

## 3. Results

**Figure 1:**
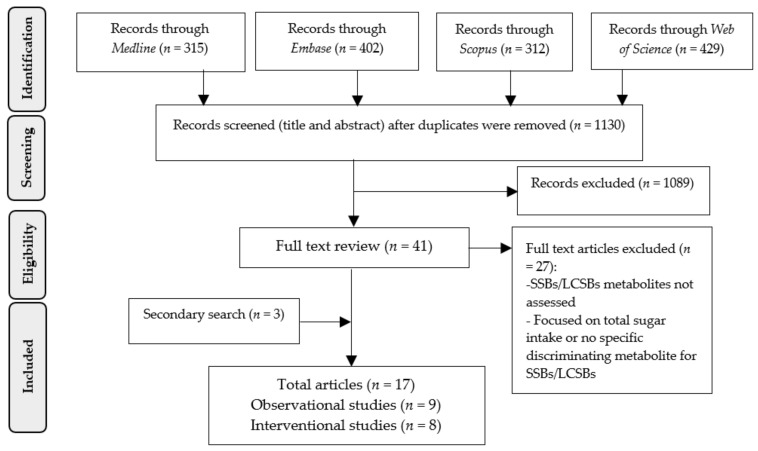
PRISMA flowchart on the screening process and selection of papers reporting biomarkers of SSBs and LCSBs as of December 2020.

The systematic literature search strategy yielded 1130 non-duplicated records from the four electronic databases. After abstract reading, full text reading for eligibility assessment, and secondary search, 17 studies were included [29,30,31,32,33,34,35,36,37,38,39,40,41,42,43,44,45], as shown in Figure 1. 

Table 1 summarizes the study characteristics and candidate biomarkers identified. There were eight cross-sectional studies [29,30,33,34,36,38,39,44,45] and eight controlled intervention studies [31,32,35,37,40,41,42,43]. For comparison with dietary intake, dietary assessment tools such as 24-h recall, 4-day and 7-day food records, and FFQS were used. Four studies were conducted in children and adolescents [35,38,39,44], two in postmenopausal women [41,42], one in predominantly obese population [31], and one study in an inpatient hospital setting [40]. Two studies used an untargeted metabolomics approach [33,39], while the rest used a targeted metabolomics approach. 

### 3.1. Carbon Isotope Based Biomarkers for SSBs Intake

The stable carbon isotope ratio ^13^C:^12^C, denoted as δ^13^C values in blood samples, plasma glucose, hair, and alanine, is significantly correlated with SSB intake [29,30,31,32,34,35,38,40,42,44,45]. Even though these studies were carried out in different settings and populations, they employed a targeted approach for biomarker discovery. Davy et al. [30] investigated the δ^13^C values of non-fasting fingerstick blood, complemented by four-day food intake records, in healthy participants. To minimize the order effects due to the sequence of dietary intake assessment, investigators randomly assigned participants to either of the two sequences for their laboratory visits, which determined the sequence of the beverage intake assessment and the four-day food intake assessment. Habitual intake of SSBs in the past month was assessed with a separate questionnaire. The δ^13^C values were correlated with the AS and SSB intakes [30]. 

Davy et al. [31], in a separate study, investigated the δ^13^C values of fasting fingerstick blood in a randomized controlled trial, using predominantly obese participants to assess whether a 6-month intervention for reducing SSBs intake was reflected on the δ^13^C values. This study concluded that, indeed, changes in δ^13^C values were associated with the AS and SSBs intake, supporting δ^13^C as an objective biomarker of AS and SSBs intake. Similarly, Fakhouri et al. [32] examined the δ^13^C values of the serum in response to an 18-month behavioral intervention program for reducing the SSBs intake in adults. Analyses of blinded serum samples confirmed the mean change in δ^13^C values, consistent with the self-reported dietary intake SSBs—further confirming earlier studies that δ^13^C values could be used to measure small changes in the intake of AS or SSBs. 

Nash et al. [45] compared the dietary intake of sugars (total, added, and SSBs) among the Yup’ik people, as reflected in the δ^13^C values in the red blood cells, hair, and fasting plasma glucose. Their dual-isotope model approach measured the values of both the δ^13^C and stable isotopes of nitrogen, δ^15^N, which controlled feeding studies have suggested as a potential biomarker for the dietary intake of meat and fish [40,41]. As other dietary components such as animal protein, honey, and beet sugar may confound the association between the AS/SSBs intake and δ^13^C values [23], there is potential utility of the dual-isotope method. Moreover, given that the δ^13^C and δ^15^N values in the red blood cells, serum, and hair have been shown to be correlated [46,47], Nash et al. [45] further examined whether the dual-isotopic model explained a similar variance in the intake of total sugars, AS, and SSBs, as reflected in the red blood cells, plasma, and hair. They observed that the three models using red blood cells, plasma, or hair isotopes explained nearly similar amounts of variance in the dietary intake of total sugar, AS, and SSBs. The strongest associations of sugar intake and δ^13^C values were observed in red blood cells and hair samples. There were strong, positive correlations in δ^13^C and δ^15^N values of red blood cells, plasma, and hair. Collectively, these results demonstrated that the δ^13^C biomarker, as reflected in red blood cells, plasma, and hair, but not in the fasting plasma glucose, may be useful in assessing the sugar intake in this Alaska Native community. 

Votruba et al. [40] used the dual-isotope model approach to measure the values of δ^13^C and δ^15^N in the red blood cells, plasma, and hair as potential biomarkers for the dietary intake of SSBs, fish, and meat in a 12-week controlled feeding trial. In this study, they observed that the δ^13^C values were significantly elevated by the dietary intake of SSBs and meat, while the δ^15^N values were significantly associated with the dietary intake of fish and meat. Specifically, the plasma δ^15^N predicted the dietary intake of fish (area under the receiver operating curve (AUC) = 0.97) and meat (AUC = 0.92), while plasma δ^13^C predicted the SSBs intake (AUC = 0.78). In all of the sample types—red blood cells, plasma, and hair—the dual-isotope approach accurately distinguished consumers of meat and fish, with a modest discrimination power for consumers of SSBs [40]. 

Two studies measured the δ^13^C of alanine as a potential biomarker of SSB intake. Choy et al. [29] investigated the association between the δ^13^C of nonessential amino acids (δ^13^C NEAA) in red blood cells and the intake of total sugar, AS, and SSBs, as well as the foods rich in animal protein such as corn-fed meats, fish, and marine mammals. Of the non-essential amino acids considered (alanine, aspartate, glutamate, glycine, proline, and serine), only the δ^13^C of alanine was strongly associated with sugar intake—total sugar, AS, and SSBs—with a moderate association between the δ^13^C of proline and SSB intake only [29]. In a subset of the study population (*n* = 30), δ^13^C of alanine in red blood cells was correlated with δ^13^C of alanine in hair samples, and intake of SSBs. As the intake of meat and fish may also elevate δ^13^C values in some populations [23,48], Choy et al. [29] further tested the specificity of the δ^13^C of alanine for SSB intake by modelling the δ^13^C of alanine as a dependent variable and adding SSBs; commercial meat, fish, and marine mammals; and intake of corn as independent variables. Unlike the findings of Votruba et al. [40], the δ^13^C values of alanine were significantly associated with SSB intake only, but not with any other dietary component, including meat and fish [29]. These findings were replicated in a recent two-week controlled feeding trial in postmenopausal women [42]. The δ^13^C of seven amino acids (alanine, glycine, valine, leucine, isoleucine, proline, and phenylalanine) in the fasting serum were measured. Like in Choy et al. [29], AS intake was associated with elevated values of δ^13^C of alanine, but was not associated with meat or any other animal protein. These two controlled studies demonstrated the specificity of δ^13^C of alanine to AS. 

Hedrick et al. [34] compared δ^13^C values of fingerstick blood with self-reported AS and SSBs intake in a cross-sectional study of adults who consumed at least 200 kcal/d from SSBs. In their multiple linear regression of δ^13^C values on other variables, they observed a significant variation in δ^13^C values across different age groups, indicating the highest intake of SSBs and AS in younger adults. Overall, SSB intake was significantly associated with δ^13^C values. Similarly, MacDougall et al. [38] explored the comparative validity, reliability, and sensitivity of δ^13^C values to reflect AS and SSBs intake in children and adolescents over a 3-week period. Their findings confirmed that δ^13^C values discriminated between high and low consumers of SSBs and between high and low consumers of AS in general (AUC = 0.75 and AUC = 0.62, respectively). In a similar study in adolescents by Liu et al. [35], but using a controlled feeding design, the δ^13^C values of the fasting fingerstick blood reflected changes in AS and ASSB intakes in different feeding periods. Valenzuela et al. [44] also focused on adolescents, and measured multiple stable isotopes, namely δ^13^C, and δ^15^N, and stable isotopes of sulfur δ^34^S, in hair and breath samples in order to evaluate the potential biomarkers for protein and carbohydrate dietary components. In this study, the intake of SSBs and C4 derived-sweets was associated with δ^13^C values from the carbon dioxide in the breath samples, both in the baseline (morning upon waking up) and post-lunch samples (1–2 h after lunch), showing the strongest correlations in the baseline samples [44]. Expectedly, significantly elevated δ^13^C values were observed among Hispanic children who were also reported to have a higher consumption of SSBs relative to non-Hispanic white children [44]. Additionally, the δ^13^C values in the hair samples were also significantly correlated with the baseline breath samples. 

Yun et al. [41] examined whole serum in postmenopausal women in a 2-week controlled diet study. This was the only study that found no association between sugar intake and δ^13^C values. Yun et al. [41] measured the values of multiple isotopes, δ^13^C, δ^15^N, and δ^34^S, in relation to the habitual intake of total sugars, AS, SSBs, animal protein, fish/seafood, red meat, dairy, poultry, and eggs. While δ^15^N predicted the intake of fish/seafood, δ^13^C moderately predicted the intake of red meat and eggs, but did not meet the biomarker threshold for the intake of sugars—total, AS, and SSBs [41]. It should be emphasized that the population in this study had limited heterogeneity in their diet, as some dietary components such as AS and SSBs were consumed in low amounts. 

### 3.2. Other Candidate Biomarkers of SSBs Intake

Some studies used untargeted metabolomics approaches to discover panels of metabolites in biosamples that could indicate the dietary intake of SSBs. Gibbons et al. [33] identified a panel of four metabolites (i.e., formate, citrulline, taurine, and isocitrate) that were significantly associated with SSB intake. They further validated these metabolites in a small acute intervention study using first-void-urine and postprandial urine samples collected at time intervals of 2, 4, and 6 h after SSB intake (i.e., a 330 mL of caloric cola). Elevated levels of the four biomarkers were recorded in the urine samples following the acute consumption of sweetened cola, and their presence was further confirmed in the chemical analysis of the cola drink [33]. 

Perng et al. [39], using an untargeted approach, also identified a novel set of metabolites associated with the intake of SSBs using fasting serum samples and an FFQ instrument for the intake assessment in children and adolescents. In this study, SSBs included non-diet sodas, fruit juices with AS, and any other beverage (e.g., tea, coffee, or water) with AS. The authors discovered sex-specific panels of biomarkers that were associated with SSB intake. They reported six biomarkers in girls—5-methyl-tetrohydrofolate, phenylephrine, urate, nonanoate, deoxyuridine, and sn-glycero-3-phosphocholine—and three biomarkers in boys—2-piperidinone, octanoylcarnitine, and catechol.

### 3.3. Candidate Biomarkers of LCSBs Intake

Three studies investigated the potential biomarkers of low-calorie sweeteners (LCSs) commonly used in LCSBs, identifying urinary excretion of acesulfame-K, saccharin, cyclamate, sucralose, and steviol glycosides among the consumers of LCSs/LCSBs [36,37,43]. Logue et al. [37] investigated the urinary excretion of commonly used LCSs following dietary exposure to LCSBs, using a double-blind, randomized crossover dose−response study. For method development and validation, participants (*n* = 12) were advised verbally and through written materials to avoid the intake of foods and beverages known to contain the five LCS compounds, at least 3 days before the 24-h urine protocol date. After the analyses, samples without concentrations of LCS (*n* = 6) were adopted for method validation. For the dose−response study, 21 participants were examined in a double-blind, randomized crossover design, lasting 3 weeks, during which participants consumed three doses of five LCSs, namely acesulfame-K, saccharin, sucralose, cyclamate, and steviol glucuronide [37]. Fasting spot and 24-h urine samples were collected at each dosing date. The 500 mL LCSBs were consumed over two consecutive days at specific times during the study period, but for the purpose of blinding the participants, 75 mL of an orange Cordial was added during LCSBs preparation. As long as the consumption did not exceed 500 mL within the 24-h period, participants were encouraged to assume normal patterns of beverage intake throughout the day. Regression analyses with the LCS dose set as the dependent variable and 24-h urinary concentrations of the LCS compounds as the independent variable explained 99% variability for acesulfame-K, 87% for saccharin, 35% for sucralose, 91% for cyclamate, and 75% steviol glucuronide [37]. These compounds were indicative of LCSBs intake. 

In a separate study, Logue et al. [36] further investigated the use of a 24-h urinary biomarker approach to detect dietary exposure to LCSB in two adult population-based studies, targeting the five LCSs investigated previously in their controlled study [37]. The 24-h urinary biomarker was compared with LCSB consumption, as self-reported in 7-d food diaries of the participants (*n* = 79), who were randomly selected from a large study regarding the prevalence of the widespread consumption of LCSs (*n* = 357). Participants were grouped into consumers and non-consumers of LCSBs on the urine protocol date. The novel urinary biomarker approach identified proportions of consumers of LCSBs enriched with various sweeteners, namely saccharin (82%), acesulfame-K (51%), cyclamates (34%), sucralose (30%), and steviol glycosides (11%) [36]. 

Sylvetsky et al. [43] investigated whether non-consumers of LCSs could be correctly characterized as unexposed using the urinary biomarker approach, in a small randomized controlled trial lasting two weeks. Participants were scheduled to attend three visits—all of which were one week apart for urine sample collection and other measurements. As they were confirmed as non-consumers of LCSs during recruitment into the study, participants were counselled to avoid dietary intake of LCSs. At baseline, their dietary intake was also recorded. After a 1-week run-in period, using sex-matched paired design, participants were randomly assigned to consume diet soda containing sucralose or unsweetened carbonated water, three times a day for a week. Other dietary components were also reviewed if they contained sucralose. At the end of the trial period, the urinary sucralose concentrations in the exposed group were consistent with the LCSB dietary intake–significantly higher than the expected residual sucralose from the occasional consumption of other dietary components containing sucralose [43].

### 3.4. Evaluation of Validity of Candidate Biomarkers

Table 2 summarizes the results of the evaluation of the candidate biomarkers for the dietary intake of SSBs. The number of times a compound is rated “Y” across validation criteria reflects the current validity of the candidate biomarker, while the “N” and “U” ratings represent areas where more research should be conducted. Candidate biomarkers δ^13^C and δ^13^C of alanine had the highest validity, with an affirmative rating on the specificity, dose−response, time−response, robustness, reliability, stability, and analytical performance. This carbon isotope ratio biomarker was also studied in many studies, consistently reporting an association with the dietary intake of SSBs or AS [29,30,31,32,34,35,38,40,42,44,45]. 

Evidence of the δ^13^C of alanine as a potential biomarker for SSBs [29,42,49] is also consistent with the long established glucose−alanine cycle in humans. The glucose−alanine cycle explains the link between carbohydrate and amino acid metabolism, in which alanine is synthesized from pyruvate, a product of glycolysis. The biochemical plausibility of δ^13^C of alanine is, therefore, demonstrable. The C4 derived AS has distinctly high δ^13^C values compared with any other dietary source, which proves the distal cause of the biomarker signal, while the proximal link between serum alanine and glucose is explained by the glucose−alanine cycle [50]. This also improves our understanding on the accumulating evidence demonstrating strong δ^13^C of alanine correlation with dietary AS, but not with other dietary components [41,42,49]. 

Uncertainty on the validity of the δ^13^C and δ^13^C of alanine as biomarkers of SSB intake remains regarding reproducibility across laboratories, as inter-laboratory results have not been described in literature. To fulfil this validation criterion, targeted analysis of the candidate biomarker in common set of samples is recommended, maintaining blind testing across testing laboratories [26]. If an untargeted metabolomics approach is used, a standardized analytical approach should be used by all participating laboratories. 

Even though formate, citrulline, taurine and isocitrate, were discovered in an observational study and were validated in a small intervention study [33], these candidate biomarkers were rated 4/9, meeting criteria for dose−response, single meal time−response, robustness, and analytical performance. Lastly, 5-methyl-tetrohydrofolate, phenylephrine, urate, nonanoate, deoxyuridine, sn-glycero-3-phosphocholine, 2-piperidinone, octanoylcarnitine, and catechol showed the lowest validity scores, with a positive rating on robustness and analytical performance only. The low scores identified areas of further research to improve the validity of these candidate biomarkers for the dietary intake of SSBs.

A summary of the evaluation of the validity of the candidate biomarkers for the dietary intake of LCSBs is provided in Table 3. Briefly, acesulfame-K, saccharin, sucralose, cyclamate, and steviol glucuronide showed moderate validity (6/9) in predicting LCSBs intake. All of these compounds are commercially used as low-calories sweeteners. As such, their plausibility as biomarkers of specific LCSBs is fulfilled, but additional qualitative assessments of the dietary intake should rule out other dietary sources. Uncertainty remains regarding their kinetics after repeated or habitual intake, as the compounds were assessed in urine, which reflects recent intake. Accumulation of the compounds as a consequence of habitual intake is inconclusive, as none of these studies investigated the usual intake. Moreover, evidence on the stability and reproducibility of these compounds in the same set of samples across various laboratories has not been described.

### 3.5. Risk of Bias and Quality of Study Assessment

The risk of bias and quality of evidence assessment for the included studies is presented in Supplementary Table S1 for the observational studies, and Supplementary Table S2 for the controlled intervention studies. Overall, the quality assessment scores for the observational studies ranged between 9 and 12.5 out of the attainable 14.5 points for this study design. Therefore, they were rated moderate to high quality. The quality scores for the interventional studies ranged between 7.5 and 9.5 out of the attainable 13 points for the controlled intervention studies. Given the high threshold for assessing the risk of bias and outcomes in controlled studies, evidence from these interventional studies was considered to be of a moderate quality.

## 4. Discussion

The main ingredient of SSBs is AS, and it is estimated that nearly half of AS is consumed through SSBs [63]. Long established biomarkers for sugar intake are 24-h urinary sucrose/fructose biomarkers [64,65,66,67] or sucrose/fructose in spot urine [68,69]. The sucrose/fructose biomarker, however, reflects the total sugar intake from all dietary components; it lacks specificity for AS and is thus not plausible for assessing SSBs intake. The carbon isotope method, demonstrated by elevated carbon isotope signatures, e.g., in urine, serum amino acids, red blood cells, or hair, reflects the dietary intake of AS [29,30,31,32,34,35,38,40,42,45]. When SSBs, which are highly correlated with AS, are consumed, the carbon isotopes are also absorbed and become available in the tissues. The δ^13^C biomarker values reflect the carbon isotopic composition of the plant from which the AS was refined, which could either be C3 or C4 photosynthetic plants [23]. For some regions, an illustrative example being the USA, sweeteners are mostly refined from corn syrup and cane sugar, which all utilize the C4 photosynthetic pathway [23]. SSBs with AS derived from C4 plants have high C4 isotope signatures. We consider this is the reason that all studies included in this review on the δ^13^C biomarker for SSBs are based on USA populations. In regions where the main source of AS is sugar beets (e.g., in Europe), which utilize C3 photosynthetic pathway, δ^13^C is not an appropriate biomarker for AS intake [6,35,50,70]. There are differential biochemical processes in C4 and C3 plants, in which C4 plants extract heavier ^13^CO_2_ from the atmosphere than C3 plants. Sugars refined from C4 plants are consequently more enriched with ^13^C isotopes. This means that the stable carbon isotope method can be applied to predict high consumers of SSBs—containing C4 derived AS—because of their elevated δ^13^C values [23,70]. 

The use of a stable carbon isotope as a biomarker of SSB intake has specific strengths. The fingerstick sample collection method is simple to conduct a minimally invasive and not burdensome task for the participants [23,70,71]. The 24-h urinary collection may be burdensome for some participants, eliciting concerns about compliance. Stable carbon isotopes of hair and breath, as shown in [44], are especially useful in large-scale epidemiologic studies. As the carbon isotopes integrate diet over a long period, typically weeks to months [40,45,72], they provide better estimates of habitual sugar intake compared with fructose and sucrose urinary biomarkers, which integrate short-term dietary intake. δ^13^C has also been shown to be stable and readily assayed in tissues such as red blood cells, serum amino acids, and human hair, for short-term and long-term exposure to AS [23,40]. As δ^13^C is more enriched in C4 photosynthetic plants compared with C3 plants, it can discriminate between AS and naturally occurring sugars (e.g., from fruits), which are mostly C3 plants. As such, beverages with high fruit concentrates are shown to have a significantly lower δ^13^C content than beverages enriched by cane sugar or corn syrup [23]. 

In a recent controlled feeding study [42], the biological plausibility for use of δ^13^C alanine as a biomarker for AS was demonstrated, as amino acid carbon isotope signatures discriminated AS from red meat/protein intake; specifically, the δ^13^C of alanine reflected a primary intake of AS. Additionally, the rest of the amino acids carbon isotope values showed an inverse association with sugar intake—total sugar, AS and SSBs—but a positive association with the intake of animal proteins and animal-derived dietary components such as red meat [42]. These findings are consistent with the results of Choy et al. [29], which demonstrated the δ^13^C of alanine of red blood cells was significantly associated with total sugar intake, AS, and SSBs, notwithstanding their differences in analytical approach, population, and dietary assessment methods. By targeting specific serum amino acids only, Yun et al. [42] further advanced the field, as previous approaches based on whole serum suggested that δ^13^C values were also associated with other dietary factors such as animal proteins sources, e.g., meat and other protein intake [41]. 

A recent study [49], not included in Table 1 because it was published outside the records search period, corroborates evidence on the specificity of δ^13^C alanine for AS and SBBs. In this study, the δ^13^C of alanine and δ^13^C of glutamate were individual predictors of SSBs intake, with a predictive accuracy of AUC ≥ 0.97 and no evident association with meat intake. The findings also suggested that using a multiple amino acids approach could improve the biomarker estimation of the SSBs intake [49]. On the other hand, the δ^13^C of essential amino acids, especially the δ^13^C of leucine, was the most promising predictor of meat intake (AUC ≥ 0.92). Moreover, an important addition of this study to the current literature was the observation that the concentration of δ^13^C of non-essential amino acids is not influenced by meat intake, reflected greater sensitivity, and was more specific to SSBs intake, unlike when the δ^13^C values of the total tissue (plasma and red blood cells) were measured [49]. Previous studies showed that δ^13^C total tissue was more strongly related to meat and/or animal protein intake than AS and SSBs [40,41]. The results of Yun et al., Choy et al., and Johnson et al. collectively validate the specificity of δ^13^C alanine as a biomarker for SSBs intake and not animal proteins [29,42,49]. Given that these three studies were conducted in diverse populations, this also demonstrates the robustness of this biomarker. What remains inconclusive from these studies is whether individual or multiple amino acid δ^13^C values best estimate AS and SSB intake, given that they used different blood fractions, derivatization, and analyses of amino acids, leading to slightly different sets of amino acids that were reliably measured [49]. 

A major limitation of the δ^13^C biomarker is its limited specificity and sensitivity with respect to AS and metabolically different sources of such sweeteners [6,38,50]. Theoretically, δ^13^C values reflect all dietary items from plants utilizing the C4 photosynthetic pathway. Hence, the biomarker may not reflect the SSBs intake alone. Moreover, the δ^13^C is limited to AS refined from C4 plants (cane sugar and corn syrup), and not sugar refined from C3 plants like the beet sugar [6,23]. Therefore, the application of the δ^13^C biomarker of SSBs is limited to populations that consume sugars refined from C4 sources. Furthermore, even though dietary glucose and fructose moieties are assumed to have similar metabolic fates, this is unpredictable and unlikely to hold true if high inter-individual variability exists [6]. Additionally, none of the studies included in this review demonstrated the validity of the δ^13^C biomarker in populations that consume a large proportion of dietary energy from corn-based foods. The values of δ^13^C in the blood samples may also be influenced by the dietary intake of meat from livestock fed corn-based diets, which potentially confounds the specificity of the δ^13^C biomarker [6,23,50]. Attempts to control this potential confounder with use of the nitrogen isotope, δ^15^N—found in proteins and not in sugar—yielded mixed findings [6,48,51], with nearly two thirds variation in the self-reported dietary intake of AS being attributed to other factors beyond the scope of the δ^13^C biomarker analysis approach [48]. The use of δ^15^N in another study only marginally increased the correlation between AS and δ^13^C values [51]. Given that the majority of studies relied on self-reported dietary data, this warrants further analyses in controlled feeding settings. However, it should be emphasized that in a recent controlled feeding study, δ^13^C in the serum amino acids rather than in whole serum or in red blood cells, was correlated with AS intake but not dietary intake of animal protein or red meat [42,49].

Even though the δ^13^C alanine biomarker for SSBs, as proposed by Choy et al. [29], is biochemically plausible and specific as validated in controlled feeding studies [40,42,49], values of the δ^13^C of alanine may be influenced by complex metabolic processes along the chain of inference, including extraneous factors such as fasting state, dietary composition, overweight, and obesity [50]. For example, the proximal link between δ^13^C of glucose and serum alanine in the glucose−alanine cycle. Additionally, because of the lack of accepted reference methods for estimating the habitual AS intake, validation studies rely on short-term controlled feeding measures, as observed in the Yun et al. study [42]. For example, they conducted a controlled feeding study for 2 weeks, yet the half-life of δ^13^C in plasma is estimated at 2.5 weeks [40]. Hence the dietary period falls short of the residence time of the serum δ^13^C of alanine [40,42]. This potentially biased the AS-δ^13^C association towards the null by attenuating the effect sizes [49]. In another study, it was determined that stable isotope ratio signatures in the plasma and red blood cells required 8–12 weeks and 15–19 weeks, respectively, to reach isotopic equilibration [40]. In the study of Johnson et al. [49], the carbon isotope ratios of the amino acids in the red blood cells were not at or near equilibrium at the end of the 12-week study. Therefore, the process of validating stable isotope biomarkers using short-term controlled feeding programs raises methodological concerns [50]. 

As for the panel of biomarkers identified in Gibbons et al. [33], none of these candidate biomarkers have been validated by another study; thus, more mechanistic investigations, besides the validation process, are warranted. Their presence in urine could be confounded by extraneous factors other than the intake of SSBs. The proposed compounds are not normally added in their pure form during the processing of cola drinks [73]. For instance, formate has been cited as an intermediate in normal metabolism, produced from different metabolic sources [53]. Taurine, commonly used as a dietary supplement in energy drinks, is also present in other food items, e.g., naturally occurring in shellfish, meat, and dairy products [55], which limits its specificity for AS. Similarly, watermelons are known to be rich dietary sources of citrulline [54]. Isocitrate, which essentially is an isomerized citrate, is used as a food additive, but dietary sources includes fruit juices, especially blackberries and vegetables such as carrots [56]. This panel of biomarkers, therefore, requires more investigations regarding their biological plausibility and robustness in other study settings. Importantly, validation study designs should account for the potential confounding effect of other dietary sources, as well as intermediates of metabolic processes that may be transformed into these candidate biomarkers. Similarly, this applies to the set of metabolites indicative of SSBs consumption in the study of Perng et al. [39]. Some of the candidate biomarkers (e.g., nonanoate) are dietary supplements and may be derived from other food groups, including fruit flavored SSBs and alcoholic drinks [39]. As none of them has been validated in intervention studies or any other general population study, their specificity and sensitivity for AS or SSBs, therefore, remains inconclusive. 

Until recently, there were almost non-existent metabolomics, population-based studies on the biomarkers of dietary LCSBs [73]. The present review identified three recent studies that have explored this research area, identifying common LCSs namely, acesulfame-K, saccharin, cyclamate, sucralose, and steviol glycosides in urine as indicative of LCSB intake. These findings support the hypothesis that a biomarker approach has potential to objectively assess the intake of common LCSBs, especially, given that most of these LCSs are excreted unchanged in urine [73]. Moreover, these compounds (i.e., acesulfame-K, saccharin, sucralose, cyclamate, and steviol glycosides) are not produced endogenously, and are highly specific to the ingestion of the parent compound [73]. However, as these LCSs are also used in other foods as sweeteners, relying on LCSBs alone as the surrogates for LCS intake may be misleading, because this biomarker approach does not discriminate specific sources of LCSs within a diet. More comprehensive methods are needed for the assessment of dietary intakes, including qualitative data and review of all foods for presence of LCSs [36]. As observed in Sylvetsky et al. [43], the presence of sucralose in urine of LCSs non-consumers confirms that people consume LCSs inadvertently in other dietary sources other than LCSBs. Other non-dietary sources of LCSs, such as personal care products (e.g., oral hygiene products), may also potentially bias the results [43,57]. Taken together, even though the urinary excretion of LCSs reflects its dietary intake [36,37], this novel approach should be further developed to account for inter-and intra-individual variations with respect to dietary intake and urinary excretions in different study settings, populations, and health status [36]. 

## 5. Conclusions

This review observed that the most promising candidate biomarker of SSBs is δ^13^C, with δ^13^C of alanine being the most robust, sensitive, and specific to SSBs. Improved estimation of the SSB intake may be realized by measuring the δ^13^C of multiple non-essential amino acids. Stable carbon isotopes in the total tissues, such as plasma and red blood cells, were observed to be confounded by other dietary components, particularly, meat, fish, and/or animal protein, therefore, showed modest discrimination power for AS and SSBs intake. A major limitation in the application of carbon isotope-based biomarkers is the inability to detect AS refined from sources that utilize the C3 photosynthetic pathway and other sources. The panel of candidate biomarkers of SSBs, as identified via untargeted metabolomics studies, require further investigation regarding their biochemical plausibility and validation in dose−response studies before they can be used in epidemiological studies. We also observed that LCSs, particularly acesulfame-K, saccharin, sucralose, cyclamate, and steviol glucuronide, may predict the intake of LCSBs in regions where such sweeteners are approved for commercial use. This is a promising area of research, as some of LCSs compounds are excreted unchanged via urine, are not produced endogenously in other metabolic processes, and are highly specific to dietary intake. However, other sweeteners may undergo metabolism into metabolites chemically indistinguishable from those produced from other dietary sources. The differences in the metabolic fates of LCSs should, therefore, be considered in biomarker discovery studies. Moreover, these sweeteners are also used in other foods. As such, the urinary concentration of these metabolites may not reflect the LCSB intake alone, unless qualitative data on other food group intake are properly assessed. In addition, given that these are urinary-based biomarkers that reflect short-term exposures, further research needs to characterize the habitual intake of LCSBs. 

## Figures and Tables

**Table 1:** Studies on the association between consumption and potential candidate biomarkers for sugar- and low-calorie sweetened beverages.

Study, Country, [Reference]	Number of Participants	Age Range (Years)	Dietary Assessment Method	Sample Type	Chemical Analytic Method	Analytic Approach	Candidate Biomarker of Food Intake/Metabolite
Davy et al. 2017, USA [31]	301	≥18	24-h recall (×3)	Fasting fingerstick blood	NA-SIMS	Targeted	δ^13^C
Choy et al. 2013, USA [29]	68	14–79	24-h recall (×4)	Red blood cells, hair	GC-IRMS	Targeted	δ^13^C–alanine
Davy et al. 2011, USA [30]	60	≥21	4-d DR	fingerstick blood	NA-SIMS	Targeted	δ^13^C
Fakhouri et al. 2014, USA [32]	144	25–79	24-h recall (×2)	Serum, after 8-h fast	IRMS	Targeted	δ^13^C
Hedrick et al. 2016, USA [34]	216	≥18	24-h recall (×3)	Fasting fingerstick blood	IRMS	Targeted	δ^13^C
Nash et al. 2014, USA [45]	68	14–79	24-h recall (×4)	Red blood cells, plasma, hair	IRMS	Targeted	δ^13^C
Votruba et al. 2019, USA [40]	32	46.2 (10.5) ^a^	7-d DR	Plasma, hair, Red blood cells	IRMS	Targeted	δ^13^C
Liu et al. 2018, USA [35]	33	12–18	24-h recall (×8)	Fasting fingerstick blood	NA-SIMS	Targeted	δ^13^C
Yun et al. 2018, USA [41] **	153	75 (4) ^a^	4-d DR	Serum	IRMS	Targeted	δ^13^C
Yun et al. 2020, USA [42]	145	75 (73, 78) ^b^	4-d DR	Serum AAs	GC-IRMS	Targeted	δ^13^C–alanine
MacDougall et al. 2018, USA [38]	126	6–11	24-h recall (×4)	Fingerstick blood	IRMS	Targeted	δ^13^C
Valenzuela et al. 2018, USA [44]	212	9–16	FFQ	Hair, Breath	GC-IRMS	Targeted	δ^13^C
Gibbons et al. 2015, Ireland [33]	565	≥18	4-d DR	Urine	H-NMR	Untargeted	Formate, citrulline, taurine, and isocitrate
Perng et al. 2019, Mexico [39]	242	8–14	FFQ	Fasting serum	LC/MS	Untargeted	Girls: 5-methyl-tetrohydrofolate, phenylephrine, urate, nonanoate, deoxyuridine, and sn-glycero-3-phosphocholine Boys: 2-piperidinone, octanoylcarnitine, and catechol
Logue et al. 2020, NL [36]	79	19–70	7-d DR	24-h urine	LC-MS	Targeted	acesulfame-K, saccharin, cyclamate, and sucralose steviol glycosides
Logue et al. 2017, NL [37]	21	25.7 (4.9) ^a^	7-d DR	Fasting spot and 24-h urine	LC-MS	Targeted	Acesulfame-K, saccharin, sucralose, cyclamate, and steviol glycosides
Sylvetsky et al. 2017, USA [43]	18	18–35	7-d DR	Spot urine	LC/MS	Targeted	Sucralose

^a^ and ^b^—values are mean (standard deviation) and median (interquartile range), respectively; δ^13^C—carbon isotope ratio biomarker, ^13^C:^12^C; AAs —amino acids; IRMS—isotope ratio mass spectrometry; GC-IRMS—gas chromatography with IRMS; NA-SIMS —natural abundance stable isotope mass spectrometry; H-NMR—proton (hydrogen) nuclear magnetic resonance; LC/MS—liquid chromatography–mass spectrometry; NL—Netherlands; 24-h recall—24 h dietary recall records; 4-d/7-d DR—4/7 day dietary records; FFQ—food frequency questionnaires. ** This study was conducted in postmenopausal women and reported negative results that, δ^13^C was not associated with an intake of sugar, both total and AS/SSBs.

**Table 2:** Evaluation of the validity of the identified candidate biomarkers for dietary intake of SSBs.

Compound/Metabolite	HMDB ID	Sample Type	Validation Criteria										
			1	2	3a	3b	4	5	6	7	8	Max. Points = 9	References
δ^13^C	-	RBCs, plasma, breath, hair	Y	Y	Y	Y	Y	Y	Y	Y	U	8	[6,23,30,31,32,34,35,38,40,41,44,45,48,51]
δ^13^C of alanine	HMDB0000161	Blood, serum, hair	Y	Y	Y	Y	Y	Y	Y	Y	U	8	[29,42,49,50,52]
Formate	HMDB0000142	Urine	N	Y	Y	U	Y	U	U	Y	N	4	[33,53]
Citrulline	HMDB0000904	Urine	N	Y	Y	U	Y	U	U	Y	N	4	[33,54]
Taurine	HMDB0000251	Urine	N	Y	Y	U	Y	U	U	Y	N	4	[33,55]
Isocitrate	HMDB0000193	Urine	N	Y	Y	U	Y	U	U	Y	N	4	[33,56]
5-Methyl-tetrohydrofolate	HMDB0001396	Serum	N	Y	U	U	Y	U	U	Y	N	2	[39]
Phenylephrine	HMDB0002182	Serum	N	U	U	U	Y	U	U	Y	N	2	[39]
Urate	HMDB0000289	Serum	N	U	U	U	Y	U	U	Y	N	2	[39]
Nonanoate	HMDB0031264	Serum	N	U	U	U	Y	U	U	Y	N	2	[39]
Deoxyuridine	HMDB0000012	Serum	N	U	U	U	Y	U	U	Y	N	2	[39]
Sn-glycero-3-phosphocholine	HMDB0000086	Serum	N	U	U	U	Y	U	U	Y	N	2	[39]
2-Piperidinone	HMDB0011749	Serum	N	U	U	U	Y	U	U	Y	N	2	[39]
Cctanoylcarnitine	HMDB0000791	Serum	N	U	U	U	Y	U	U	Y	N	2	[39]
Catechol	HMDB0240490	serum	N	U	U	U	Y	U	U	Y	N	2	[39]

SSBs—sugar-sweetened beverages; RBCs – red blood cells; Y—yes; N—no; U—unknown/uncertain (validation criteria adapted from [26]). 1: Plausibility—Is the marker compound plausible as a specific BFI for the food or food group (chemical/biological plausibility)? 2: Dose Response—Is there a dose−response relationship at the relevant intake levels of the targeted food (quantitative aspect)? 3: Time Response—(a) Single dose: meal time−response relationship of the BFI has been described for a defined sample type and time window in a meal study. (b) Multiple doses: the kinetics of the BFI after repeated intakes has been described for a defined sample type in a meal study or the accumulation of BFI in certain sample types has been observed. Is the biomarker kinetics for the repeated intake of the food/food group described adequately providing the frequency of sampling needed to assess the habitual intake (e.g., cumulative aspects). 4: Robustness—Has the marker been shown to be robust after the intake of complex meals reflecting the dietary habits of the targeted population? 5: Reliability—Has the marker been shown to compare well with other markers or questionnaire data for the same food/food group (reliability)? 6: Stability—Is the marker chemically and biologically stable during bio specimen collection and storage, making measurements reliable and feasible? 7: Analytical Performance—Are analytical variability, accuracy, sensitivity, and specificity known to be adequate for at least one reported analytical method? 8: Reproducibility—Has the analysis been successfully reproduced in another laboratory?

**Table 3:** Evaluation of the validity of the identified candidate biomarkers for the dietary intake of LCSBs.

Compound/Metabolite	HMDB ID	Sample Type	Validation Criteria										
			1	2	3a	3b	4	5	6	7	8	Max. Points = 9	References
Acesulfame-K	HMDB0033585	Urine	Y	Y	Y	U	Y	Y	U	Y	U	6	[36,57]
Saccharin	HMDB0029723	Urine	Y	Y	Y	U	Y	Y	U	Y	U	6	[36,37,58]
Cyclamate	HMDB0031340	Urine	Y	Y	Y	U	Y	Y	U	Y	U	6	[36,37,57,59,60]
Sucralose	HMDB0031554	Urine	Y	Y	Y	U	Y	Y	U	Y	U	6	[36,43,57,61]
Steviol glycosides	HMDB0036707	Urine	Y	Y	Y	U	Y	Y	U	Y	U	6	[14,37,57,62]

LCSBs—low-calorie sweetened beverages; Response: Y—yes; N—no; U—unknown/uncertain (validation criteria adapted from [26], as explained under Table 2).

## Supplementary Materials

The following are available online at https://www.mdpi.com/article/10.3390/metabo11080546/s1. Table S1: Combined quality assessment using NUTRIGRADE and BIOCROSS tools for evaluation of observational studies and biomarkers in human research, Table S2: Combined quality assessment using NUTRIGRADE and BIOCROSS tools for evaluation of controlled intervention studies and biomarkers in human research.
