# Supplementary material for: A Systematic Review of Metabolomic Biomarkers for the Intake of Sugar-Sweetened and Low-Calorie Sweetened Beverages
Source: Metabolites. 2021 Aug 19;11(8):546. doi: 10.3390/metabo11080546 (PMC8401376; doi:10.3390/metabo11080546)
Supplement: Supplementary file 1 [file metabolites-11-00546-s001.zip › metabolites-1309686-supplementary.pdf]

Table S1: Combined quality assessment using NUTRIGRADE and BIOCROSS tools for evaluation of observational studies and biomarkers in human research

Study	Evaluation of the study					Evaluation of Biomarker assessment and reporting			Total rating* (Max. 14.5 points)
	Risk of bias, study quality (0-1.5)	Study limitations (0-2)	Statistical Analysis (0-2)	Data interpretation (0-2)	Funding bias (0-1)	Specimen & assay methods (0-2)	Laboratory measurement (0-2)	Biomarker data modelling (0-2)	
**Choy et al. 2013**	1.5	2.0	1.0	2.0	1.0	2.0	1.0	1.0	11.5
**Davy et al. 2011,**	0.5	1.0	0.5	2.0	1.0	2.0	1.0	1.0	9.0
**Hedrick et al. 2016**	1.5	1.0	1.0	2.0	1.0	2.0	1.0	1.0	10.5
**Nash et al. 2014**	1.5	2.0	1.0	2.0	1.0	2.0	1.0	1.0	11.5
**MacDougall et al. 2018**	1.5	2.0	1.0	2.0	1.0	2.0	1.0	1.0	11.5
**Gibbons et al. 2015**	1.5	1.0	1.0	2.0	1.0	2.0	1.0	2.0	11.5
**Perng et al. 2019**	1.5	2.0	2.0	2.0	1.0	2.0	1.0	2.0	12.5
**Valenzuela et al. 2018**	1.0	1.0	1.0	2.0	1.0	2.0	1.0	1.0	10.0
**Logue et al. 2020**	1.0	1.0	1.0	2.0	1.0	2.0	1.0	1.0	10.0

Table S2: Combined quality assessment using NUTRIGRADE and BIOCROSS tools for evaluation of controlled intervention studies and biomarkers in human research

Study	Evaluation of the study				Evaluation of Biomarker assessment and reporting			Total Rating* (Max. 13 points)
	Risk of bias, study quality (0-3)	Precision (0-1)	Funding bias (0-1)	Study design (0 or 2)	Specimen & methods (0-2)	Laboratory measurement (0-2)	Biomarker data modelling (0-2)	
**Davy et al. 2017**	1.5	0	1.0	2.0	2.0	1.0	1.0	8.5
**Fakhouri et al. 2014**	2.0	0	1.0	2.0	2.0	1.0	1.0	9.0
**Votruba et al. 2019**	2.5	0	1.0	2.0	2.0	1.0	1.0	9.5
**Liu et al. 2018**	1.5	0	1.0	2.0	2.0	1.0	1.0	8.5
**Yun et al. 2018**	0.5	0	1.0	2.0	2.0	1.0	1.0	7.5
**Yun et al. 2020**	0.5	0	1.0	2.0	2.0	1.0	1.0	7.5
**Logue et al. 2017**	1.5	0	1.0	2.0	2.0	1.0	1.0	8.5
**Sylvetsky et al. 2017**	1.0	0	1.0	2.0	2.0	1.0	1.0	8.0

For both Table S1 and S2: *Scores of 0–5 were considered low quality, 5–10 moderate quality and scores ≥ 10 points were considered high quality.
